# Impacts of Annealing Conditions on the Flat Band Voltage of Alternate La_2_O_3_/Al_2_O_3_ Multilayer Stack Structures

**DOI:** 10.1186/s11671-016-1623-2

**Published:** 2016-09-13

**Authors:** Xing-Yao Feng, Hong-Xia Liu, Xing Wang, Lu Zhao, Chen-Xi Fei, He-Lei Liu

**Affiliations:** Key Laboratory for Wide-Band Gap Semiconductor Materials and Devices of Education, School of Microelectronics, Xidian University, Xi’an, 710071 China

**Keywords:** Flat band voltage, La_2_O_3_/Al_2_O_3_ multilayer, Dipoles, Diffusion effect, Interface layer

## Abstract

The mechanism of flat band voltage (V_FB_) shift for alternate La_2_O_3_/Al_2_O_3_ multilayer stack structures in different annealing condition is investigated. The samples were prepared for alternate multilayer structures, which were annealed in different conditions. The capacitance-voltage (C-V) measuring results indicate that the V_FB_ of samples shift negatively for thinner bottom Al_2_O_3_ layer, increasing annealing temperature or longer annealing duration. Simultaneously, the diffusion of high-*k* material to interfaces in different multilayer structures and annealing conditions is observed by X-ray photoelectron spectroscopy (XPS). Based on the dipole theory, a correlation between the diffusion effect of La towards bottom Al_2_O_3_/Si interface and V_FB_ shift is found. Without changing the dielectric constant *k* of films, V_FB_ shift can be manipulated by controlling the single-layer cycles and annealing conditions of alternate high-*k* multilayer stack.

## Background

High dielectric constant (high-*k*) materials have been extensively used to substitute conventional SiO_2_ gate oxides for its prominent properties such as small equivalent oxide thickness (EOT) and low leakage current. During the past years, researchers have paid lots of attentions to high-*k* materials, such as hafnium oxide (HfO_2_), yttrium oxide (Y_2_O_3_), zirconium oxides (ZrO_2_), lanthanum oxide (La_2_O_3_), aluminum oxide (Al_2_O_3_), and other transition-metal oxides. Among them, La_2_O_3_ is considered a remarkable candidate because of its high dielectric constant (approximately 25) and large band gap (approximately 5.8 eV). However, the application of high-*k* materials also cause lots of new problems and challenges [1, 2]. Recently, the properties of La_2_O_3_ and Al_2_O_3_ gate stacks have been studied by many researchers, and much promotion has been made in restraining leakage current and suppressing the formation of parasitic interface [3–5].

Furthermore, flat band voltage has been regarded as one of the most critical parameters for the design and fabrication of semiconductor devices. Earlier researchers claimed that the fixed charges are the main factor for flat band voltage (V_FB_) shift [6]. However, Dr. Wang pointed out there was no correlation between V_FB_ and fixed charges because the film Hf_*x*_La_1 − *x*_O_*y*_ keep the same V_FB_ for different film thicknesses [7, 8]. Researchers also revealed that the main origin of V_FB_ is the dipoles between high-*k*/interface layer [9, 10]. Besides, the atomic mechanism of V_FB_ shifts for different high-*k* gate stacks is also discussed specifically by Lin and Robertson [11, 12]. However, the influence of the film structure and annealing conditions on V_FB_ has not been fully investigated. In this study, firstly, a model of V_FB_ including the interfaces of metal/high-*k* and high-*k*/Si was introduced. Then, alternate La_2_O_3_/Al_2_O_3_ multilayer stacks were prepared with different single-layer cycles by atomic layer deposition (ALD) and annealed in different temperatures and duration. The electrical and physical characteristics of the samples were investigated. Based on the theory of dipoles and diffusion effect, the mechanism of V_FB_ shift was studied.

## Methods

Firstly, p-type Si(100) wafers were washed in deionized water and chemically etched with diluted HF for 3 min to remove the native oxide. Then, alternate La_2_O_3_/Al_2_O_3_ multilayer high-*k* stacks with different single-layer cycles were deposited on Si wafers by ALD reactor (Picosun R-150, Espoo, Finland) in 300 °C. La(^i-^PrCp)_3_ and trinethyluminium (TMA) were used as precursors of La and Al, respectively. Besides, O_3_ was used as oxidant, and ultra-high purity nitrogen (N_2_, 99.999 %) was employed as carrier and purge gas. After deposition, the rapid thermal annealing (RTA) process was carried out at different temperatures for different duration in N_2_ ambient. For further analysis, annealed La_2_O_3_/Al_2_O_3_ film thickness (without metal gate) was examined by Woollam M2000D (Woollam Co. Inc., Lincoln, NE, USA) spectroscopic ellipsometry (SE). X-ray photoelectron spectroscopy (XPS) was used to examine the bonding structures and chemical quantitative composition of the films. C1s peak from adventitious carbon at 284.6 eV [13] was used as an internal energy reference during the analysis. Besides, 100-nm-thick Al was deposited by magnetron sputtering as electrode, and then, capacitance-voltage (C-V) measurement was carried out using Agilent B1500A semiconductor analyzer at the frequency of 100 kHz.

## Results and Discussion

Taking into consideration of fixed charges and interfacial dipoles, the V_FB_ of conventional metal/SiO_2_/Si metal oxide semiconductor (MOS) structure can be expressed as [14]:

1 $$ {V}_{\mathrm{FB}}=\frac{\varphi_{\mathrm{ms}}}{q}-\mathrm{E}\mathrm{O}\mathrm{T}\left(\frac{Q_0}{\varepsilon_0{\varepsilon}_{\mathrm{ox}}}\right)+\left({\varDelta}_{{\mathrm{metal}/\mathrm{S}\mathrm{i}\mathrm{O}}_2}+{\varDelta}_{{\mathrm{SiO}}_2/\mathrm{S}\mathrm{i}}\right), $$

where *φ*_ms_ is the work function difference between metal and Si substrate and *Q*_0_ represents the fixed charges located in oxide layer. Δmetal/SiO_2_ and ΔSiO_2_/Si are dipoles located in the interface of metal/SiO_2_ and SiO_2_/Si.

In this work, SiO_2_ is substituted by high-*k* materials Al_2_O_3_ and La_2_O_3_, so the V_FB_ of samples with alternate high-*k* dielectric gate stacks can be expressed as:

2 $$ {V}_{\mathrm{FB}}=\frac{\varphi_{\mathrm{ms}}}{q}-{d}_0\left(\frac{Q_0}{\varepsilon_0{\varepsilon}_{{\mathrm{Al}}_2{\mathrm{O}}_3}}\right)-{d}_1\left(\frac{Q_1}{\varepsilon_0{\varepsilon}_{{\mathrm{La}}_2{\mathrm{O}}_3}}\right)+\left({\varDelta}_{\mathrm{metal}/\mathrm{high}\hbox{-} k}+{\varDelta}_{\mathrm{high}\hbox{-} k/\mathrm{S}\mathrm{i}}+{\varDelta}_{{\mathrm{La}}_2{\mathrm{O}}_3/{\mathrm{Al}}_2{\mathrm{O}}_3}\right). $$

**Fig. 1:**
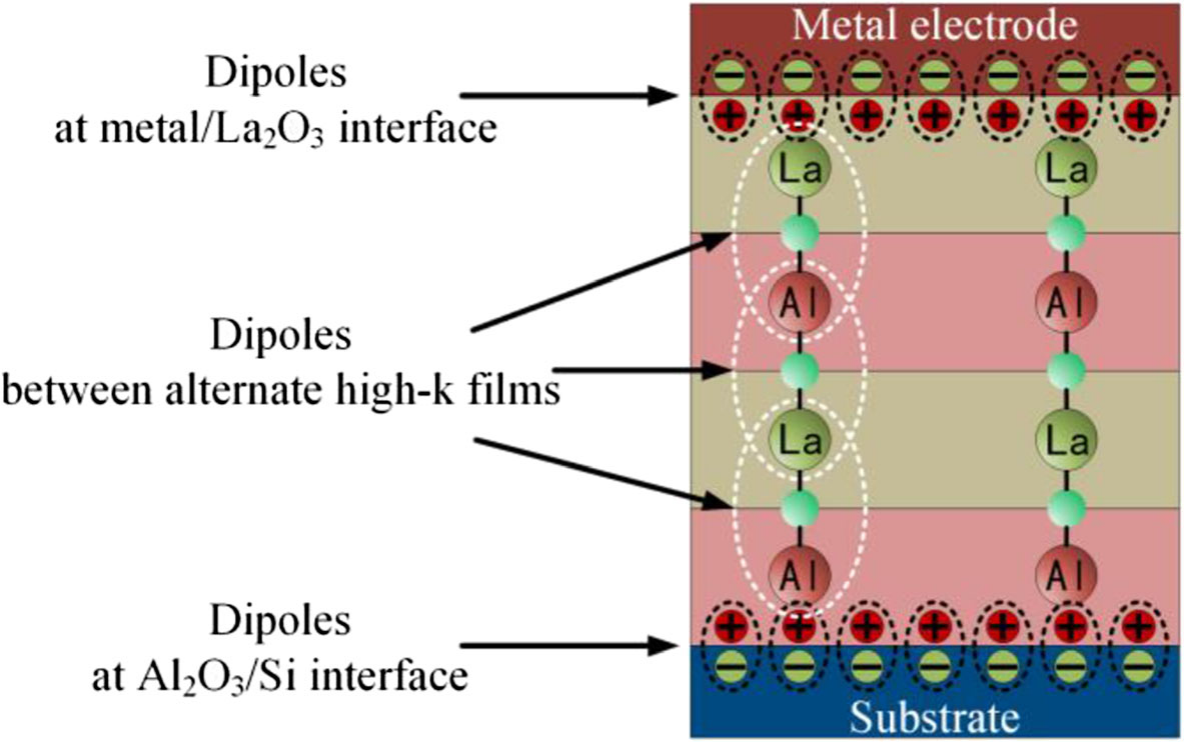
The schematic of three kinds of dipoles in La_2_O_3_/Al_2_O_3_ high-*k* dielectric stacks

In this equation, *Q*_0_ and *Q*_1_ represent the fixed charges in Al_2_O_3_ and La_2_O_3_ films, respectively. As shown in Fig. 1, all dipoles can be separated into three kinds. They are the dipoles between the alternate high-*k* films, the dipoles at interfaces of metal/La_2_O_3_, and dipoles at interfaces of Al_2_O_3_/Si. Between the alternate high-*k* layers, the dipoles La–O–Al and Al–O–La have reverse sequence which can cancel out each other. So these dipoles do not create net dipole.

**Fig. 2:**
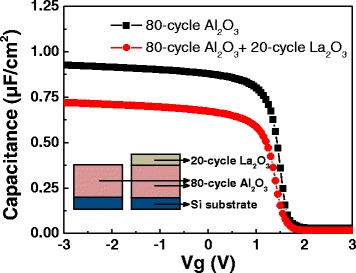
The simplified schematic structures and C-V curves of La_2_O_3_/Si and Al_2_O_3_/La_2_O_3_/Si

Furthermore, some researches have proved that the contribution of dipoles at metal/high-*k* interface to the V_FB_ shift can be neglected [10, 15, 16]. For inspecting this point of view, two samples were prepared with different high-*k* films, and then, the RTA process was carried out at 600 °C for 60 s in N_2_ atmosphere. Their simplified schematic structures and C-V curves are shown in Fig. 2. It should be noted that the two films show approximately the same V_FB_: 1.49 V (without La_2_O_3_ inserted layer) and 1.47 V (with La_2_O_3_ inserted layer). The insensitiveness of V_FB_ values to the kind of dipoles at metal/high-*k* interface clearly indicates the metal/high-*k* interface is not one of the origin of V_FB_ shift. Therefore, in this work, the fixed charges *Q*_0_ and *Q*_1_ and the dipoles at interface of Al_2_O_3_/Si need to be examined on the next step.

**Fig. 3:**
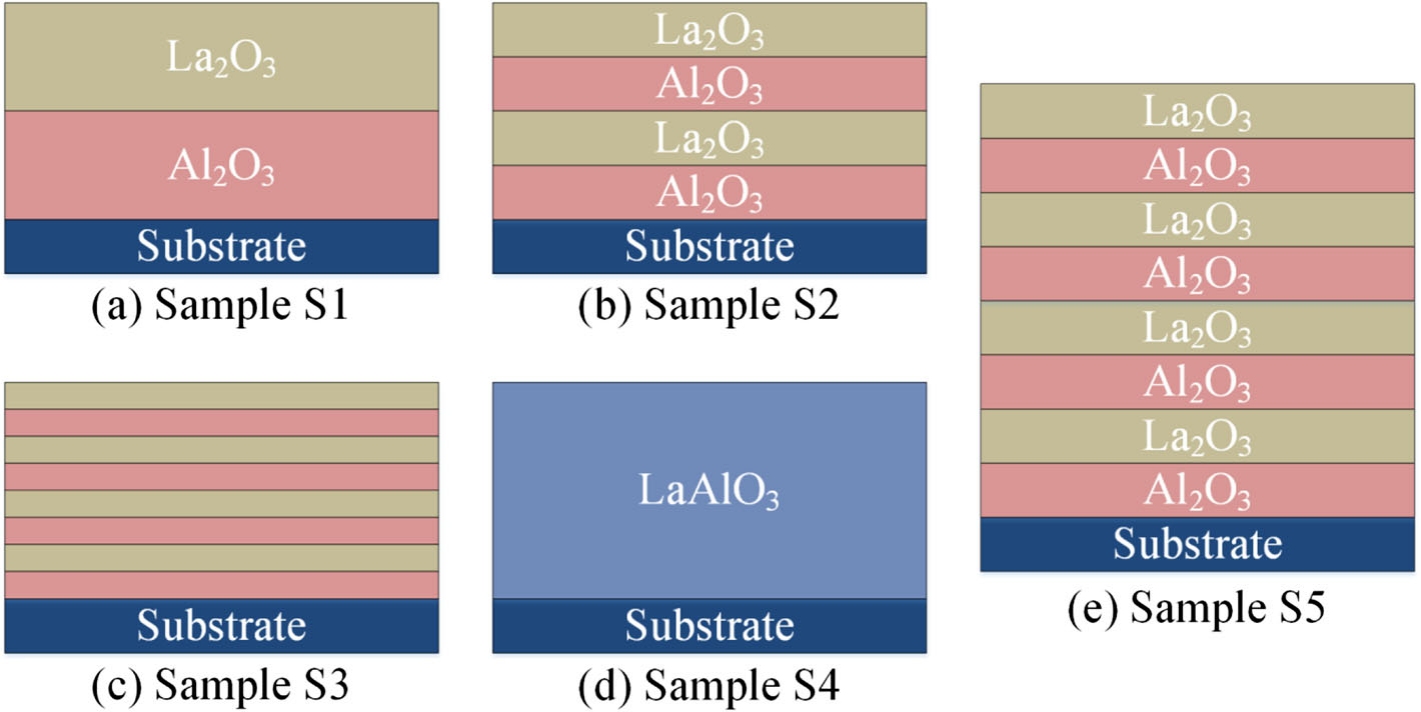
The schematic structures of samples S1~S5

Samples S1~S5 were deposited for different structures in an identical annealing condition (annealed at 600 °C for 60 s in N_2_ atmosphere). The schematic of alternate high-*k* films S1~S5 is shown in Fig. 3. For each of the samples S1~S4, 40-cycle La_2_O_3_ and 40-cycle Al_2_O_3_ were deposited with different single-layer cycles (from S1 to S4 are 40, 20, 10, and 1 cycle of single layer). In order to investigate the influence of fixed charges on V_FB_, as shown in Fig. 3b, e, the sample S5 was deposited with the same single-layer cycles but double number of layers as S2. The structures and thicknesses of samples S1~S5 are listed in Table 1.

**Table 1:** The structures and thicknesses of annealed samples S1~S5

Sample	Film structures	Thickness (nm)
S1	1 × (40-cycle Al_2_O_3_ + 40-cycle La_2_O_3_)	7.06
S2	2 × (20-cycle Al_2_O_3_ + 20-cycle La_2_O_3_)	7.02
S3	4 × (10-cycle Al_2_O_3_ + 10-cycle La_2_O_3_)	6.99
S4	40 × (1-cycle Al_2_O_3_ + 1-cycle La_2_O_3_)	7.57
S5	4 × (20-cycle Al_2_O_3_ + 20-cycle La_2_O_3_)	13.4

The samples were annealed at 600 °C for 60 s

**Fig. 4:**
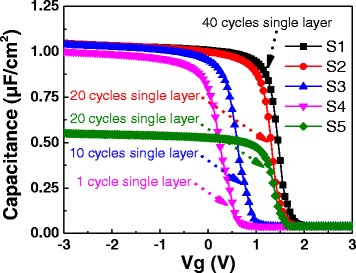
C-V curves of samples S1~S5 which contain 40, 20, 10, 1, and 20 single-layer cycles, respectively

**Fig. 5:**
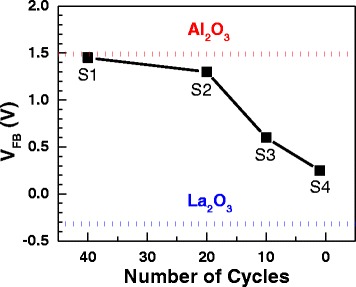
V_FB_ of samples S1~S5. The *red* and *blue dotted lines* represent the V_FB_ of 80-cycle Al_2_O_3_ and La_2_O_3_ films, respectively

Figures 4 and 5 show the C-V curves and V_FB_ shifts of samples S1~S5. Samples S1~S4 have very close accumulation capacitance. The EOTs of samples S1~S5 are extracted by NCSU CVC program [17], which are 2.21, 2.20, 2.21, 2.29, and 4.16 nm, respectively. The dielectric constants are 12.46, 12.44, 12.34, 12.89, and 12.56, respectively. The V_FB_ of samples S1~S5 are 1.45, 1.30, 0.60, 0.25, and 1.30 V, respectively. For 80-cycle pure Al_2_O_3_ and 80-cycle pure La_2_O_3_ films deposited and annealed in the same condition with S1~S5, V_FB_ are 1.49 and −0.32 V, which is shown in Fig. 5. We notice that V_FB_ become smaller shifting from the V_FB_ of pure Al_2_O_3_ film to the pure La_2_O_3_ direction for a thinner single layer (from 40 to 1 cycle). In a recent report [10], a negative V_FB_ shift is observed for a thicker La_2_O_3_ inserted layer at HfO_2_/Si interface which comes to a similar conclusion with our work. Furthermore, V_FB_ is the same for samples S2 and S5, which indicates the Al_2_O_3_ and La_2_O_3_ films have few fixed charges. Therefore, the fixed charge *Q*_0_ and *Q*_1_ in Eq. 2 can be neglected for studying V_FB_ shift. As discussed, the V_FB_ shifts of S1~S4 have no relevance to the dipoles between alternate high-*k* layers and dipoles at metal/high-*k* interface. Therefore, it is clear that such a shift (from 1.45 to 0.25 V) is supposed to be relevant to the variation of dipoles at Al_2_O_3_/Si interface.

**Fig. 6:**
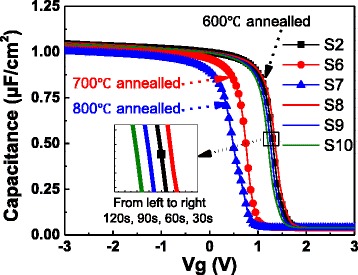
C-V curves of samples S2 and S6~S10. Samples S2, S6, and S7 were annealed at different temperatures for 60 s, while samples S2 and S8~S10 were annealed at 600 °C for different duration

**Fig. 7:**
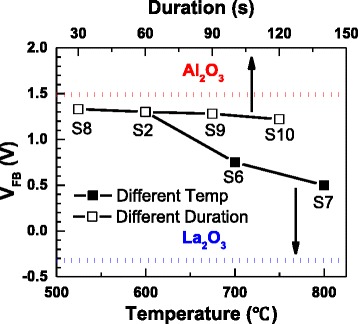
V_FB_ of samples S2 and S6~S10. S2 is a shared point for different annealing temperature and duration. The *red* and *blue dotted lines* represent the V_FB_ of 80-cycle Al_2_O_3_ and La_2_O_3_ films, respectively

For further investigation about the mechanism of V_FB_ shift for high-*k* gate stacks, different annealing conditions were employed after the ALD deposition. As shown in Table 2, samples S2 and S6~S10 were deposited for identical structure and then annealed at different temperatures (600, 700, and 800 °C) for different duration (30, 60, 90, and 120 s) in N_2_ atmosphere. The correlations between C-V curves and annealing conditions of samples S2 and S6~S10 are shown in Fig. 6. The EOTs of samples S2 and S6~S10 extracted by NCSU CVC program [17] are 2.20, 2.23, 2.29, 2.20, 2.20, and 2.20 nm, respectively, and the dielectric constant *k* can be figured out as 12.44, 12.61, 12.26, 12.55, 12.53, and 12.62, respectively. As shown in Fig. 7, the V_FB_ of samples S2 and S6~S10 are 1.3, 0.75, 0.5, 1.33, 1.28, and 1.22 V, respectively, which have a remarkable negative shift with increasing annealing temperature and a slight negative shift with increasing duration. Similar trend of V_FB_ shift (approximately 1 to 0.6 V) was also reported for HfO_2_ and Al_2_O_3_ stacks at different annealing temperature (400 and 1000 °C, respectively) [9].

**Table 2:** The annealing conditions and thicknesses of samples S2 and S6~S10

Sample	Temperature (°C)	Duration (s)	Thickness (nm)
S2	600	60	7.02
S6	700	60	7.21
S7	800	60	7.20
S8	600	30	7.08
S9	600	90	7.07
S10	600	120	7.12

Samples S6~S10 have the same film structure with S2: 2 × (20-cycle Al_2_O_3_ + 20-cycle La_2_O_3_)

**Fig. 8:**
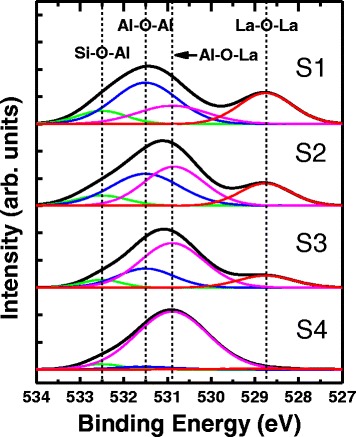
O1s XPS spectra of samples S1~S4. The O1s spectra were fitted with four peaks (Si–O–Al, Al–O–Al, Al–O–La and La–O–La)

Then, XPS was employed to examine the variation of bonding structure. Figure 8 shows the O1s XPS spectra of annealed samples S1~S4, and each of the spectra was fitted with four peaks Si–O–Al (532.5 eV), Al–O–Al (531.5 eV), Al–O–La (530.9 eV), and La–O–La (528.75 eV). It is found that La–O–Al peaks become larger while Al–O–Al and La–O–La peaks become smaller from S1 to S4. That is attributed to more La_2_O_3_/Al_2_O_3_ interface layers formed with decreasing single-layer cycles. As we discussed above, the dipoles La–O–Al and Al–O–La can cancel each other, so the variation of these peaks makes no contribution to V_FB_ shift.

**Fig. 9:**
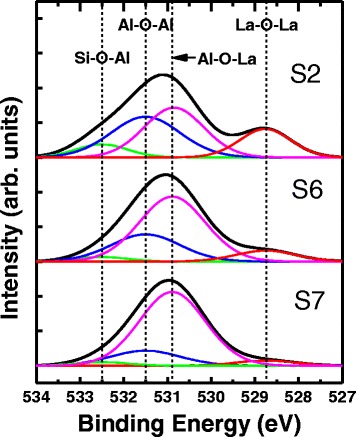
O1s XPS spectra of samples S2, S6, and S7. The O1s spectra was fitted with four peaks (Si–O–Al, Al–O–Al, Al–O–La and La–O–La)

Figure 9 show the O1s XPS spectra of samples S2, S6, and S7. More Al–O–La bonds and less Al–O–Al and La–O–La bonds are observed from S2 to S7, which indicates more Al_2_O_3_ and La_2_O_3_ diffuse into each other and form LaAlO_3_ at Al_2_O_3_/La_2_O_3_ interface at higher annealing temperature. The value of diffusion coefficient mainly depends on the kinds of diffusion substance and diffusion medium as well as the diffusion temperature. So this trend is due to the larger diffusion coefficient obtained at higher temperature.

In both Figs. 8 and 9, we notice that there is only small amount of Si–O–Al bonds which show a slight decrease for thinner single layer or higher annealing temperature. Thinner single layer means a thinner bottom Al_2_O_3_ layer, which leads to more La_2_O_3_ diffusing into Al_2_O_3_/Si interface and replacing the Si–O–Al with Si–O–La bonds. Based on the theory of diffusion, more La_2_O_3_ can diffuse through the bottom Al_2_O_3_ layer forming Si–O–La bonds at higher annealing temperature. Similarly, increasing annealing duration can also cause more La_2_O_3_ diffusing into Al_2_O_3_/Si interface. That is why, the amount of Si–O–Al bonds declines. This diffusion effect of high-*k* material after annealing process is also proved in report [9], which shows HfO_2_ and Al_2_O_3_ stacks diffusing into each other and into the metal/high-*k* and high-*k*/Si interfaces after annealing process at different temperatures.

On the other hand, Si–O–Al and Si–O–La bonds are located at the interface of high-*k*/Si. According to the dipole theory discussed above, this substitution of La for Al should be responsible for the negative V_FB_ shift. Therefore, we should also discuss how the increasing La–O–Si bonds at Al_2_O_3_/Si interface influences the V_FB_. La has weaker electronegativity than Al (La ~ 1.11, Al ~ 1.61). When Al is substituted by La, compared with Al, electrons will be further from La and move towards O. So the dipole La–O presents a larger polarity compared with dipole Al–O. It means a larger electrostatic potential, which can increase the band offset and finally cause the V_FB_ shift. It is concluded that the more Al is substituted by La at Al_2_O_3_/Si interface the closer to blue dotted line, the V_FB_ will be. Moreover, the V_FB_ shift in different annealing temperatures is much bigger compared with different duration. It can be explained by the exponential relationship between the temperature and diffusion coefficient. Therefore, a feasible way to modulate the V_FB_ of alternate high-*k* multilayer stack gate is to control the single-layer cycles and annealing conditions.

In addition, we should notice that the diffusion should be bidirectional, meaning the Al_2_O_3_ should also diffuse into the metal/La_2_O_3_ interface. But the experiment in our work shows no relevance between the metal/high-*k* interface and the V_FB_ shift. In fact, some researchers hold the contrary opinions to our work by investigating the high-*k* inserted layer at metal/high-*k* interface. However, in these reports [18, 19], the high temperature annealing (1000 °C) and thin high-*k* films (approximately 4 nm) may lead to the non-negligible diffusion of inserted layer material to the high-*k*/Si interface and result in V_FB_ shift. Furthermore, the reason that dipoles at metal/high-*k* and high-*k*/Si interface present distinct effect on V_FB_ shift is supposed to be relevant to the different properties of bonds. Unlike La–O–Si or Al–O–Si, the bonds between metal gate and high-*k* material are ionic bonding (La–O–Al or Al–O–Al), which may result in distinctly different electrical properties of dipoles. However, further work is still underway towards a more specific explanation about that.

## Conclusions

The C-V curves and XPS results of alternate La_2_O_3_/Al_2_O_3_ multilayer stacks are investigated in the paper. It is concluded that the main factor of V_FB_ shift is the dipoles at high-*k*/Si interface. Furthermore, the V_FB_ of samples shifts negatively and varies from the V_FB_ of pure Al_2_O_3_ to the pure La_2_O_3_ direction for thinner bottom Al_2_O_3_ layer, increasing annealing temperature or longer annealing duration. In such a condition, more La_2_O_3_ can diffuse into the Al_2_O_3_/Si interface and form La–O–Si bonds. Because of a weaker electronegativity of La, dipole La–O has stronger polarity than dipole Al–O. It leads to the band offset and negative V_FB_ shift. As a result, a feasible way to modulate V_FB_ without changing the dielectric constant *k* of films is proposed.

## Abbreviations

ALD: Atomic layer deposition
C-V: Capacitance-voltage
EOT: Equivalent gate oxide thickness
MOS: Metal oxide semiconductor
RTA: Rapid thermal annealing
SE: Spectroscopic ellipsometry
TMA: Trinethyluminium
V_FB_: Flat band voltage
XPS: X-ray photoelectron spectroscopy
